# Electroencephalographic and Cardiovascular Assessments of Isoflurane-Anesthetized Dogs

**DOI:** 10.3390/vetsci11100514

**Published:** 2024-10-18

**Authors:** Jeff C. Ko, Carla Murillo, Ann B. Weil, Matthias Kreuzer, George E. Moore

**Affiliations:** 1 Department of Veterinary Clinical Sciences, College of Veterinary Medicine, Purdue University, West Lafayette, IN 47907, USA; murilloc@purdue.edu (C.M.); aweil@purdue.edu (A.B.W.); 2 Department of Anesthesiology and Intensive Care, School of Medicine and Health, Technical University of Munich, 80333 München, Germany; m.kreuzer@tum.de; 3 Department of Veterinary Administration, College of Veterinary Medicine, Purdue University, West Lafayette, IN 47907, USA; gemoore@purdue.edu

**Keywords:** isoflurane, electroencephalography, dogs, cardiorespiratory, antinociception, minimum alveolar concentration, patient state index, burst suppression ratio, blood pressure, heart rate, unconsciousness, unresponsive

## Abstract

This study investigated the use of frontal electroencephalography (EEG) to monitor varying levels of isoflurane anesthesia in dogs. The patient state index (PSI), burst suppression ratio (SR), and waveforms, were continuously recorded while mean arterial blood pressure (MBP), heart rate, responses to electric stimuli, and subjective anesthetic “depth” were assessed every 3 min. At deep anesthesia (2.5× MAC − 3.2%), the PSI (6.5 ± 10.8) and MBP (45.6 ± 16.4 mmHg) were the lowest, and SR was the highest (78.3 ± 24.0%). At 1× MAC (1.3%), the PSI and MBP increased significantly to 47.8 ± 12.6 and 99.8 ± 13.2, respectively, and SR decreased to 0.5 ± 2.5%. The EEG was predominantly isoelectric at 2×–2.5× MAC, indicating unconsciousness and unresponsiveness. As anesthesia lightened, waveforms transitioned to flatter and faster activity patterns with a response to noxious stimuli, suggesting regained consciousness. The PSI and MBP exhibited a stronger correlation (ρ = 0.8098, *p* = 0.001) than the relationship of PSI with heart rate (ρ = −0.2089, *p* = 0.249). Five of the six dogs experienced rough recovery, possibly due to high SR and low MBP. These findings suggest that EEG monitoring in dogs can be a valuable tool for the real-time tracking of brain states and can be used to guide the management of isoflurane anesthesia.

## Simple Summary

This study investigated the application of frontal electroencephalography (EEG) to monitor isoflurane anesthesia in dogs. By varying anesthetic levels from deep to light planes, we observed distinct changes in EEG waveforms. At deep anesthesia (2–2.5× MAC-minimum alveolar concentration), EEG patterns were predominantly isoelectric with rare intermittent bursts. The dog was unresponsive to electrical stimulation, indicating complete unconsciousness. As anesthesia lightened (1–1.5× MAC), EEG patterns transitioned to alpha and beta dominance with occasional burst suppression. Responsiveness to electrical stimulation signified the recovery of consciousness. At light anesthesia (0.75× MAC) and recovery, EEG amplitudes decreased, and the frequency increased. This study found a stronger correlation between mean arterial blood pressure and the patient state index, a processed EEG parameter, compared to heart rate. These findings suggest that EEG is a valuable tool for the real-time monitoring and management of isoflurane anesthesia levels in dogs.

## 1. Introduction

Clinical practice traditionally relies heavily on cardiovascular parameters such as heart rate (HR) and blood pressure to assess the level of anesthesia [1,2,3,4]. However, the reliability of these measures may be limited by several factors. Various intrinsic and extrinsic factors, including cardiac medications, vasoactive agents, hydration status, and underlying comorbidities, can significantly influence these readings. Consequently, these parameters may not accurately reflect changes in brain state, potentially delaying the recognition of critical alterations in the level of anesthesia. Moreover, individual patient variability in response to anesthesia further complicates the interpretation of these hemodynamic data [5,6,7,8,9,10]. This highlights the need for more direct methods to monitor anesthesia. Recent developments in the field of anesthesia monitoring are actively exploring such approaches for both humans and animals [5,6,7,8,11]. One promising technology is frontal electroencephalography (EEG), which offers a real-time window into the brain state perioperatively [5,7,8,9,10]. Studies in humans have shown distinct EEG pattern changes that correlate with different concentrations of inhalational anesthetics like isoflurane during surgery [5,6,7,8]. The use of frontal EEG with advanced algorithms has also been investigated in pigs [11], horses [12,13,14], and dogs [15,16,17,18,19]. However, despite prior attempts in dogs, there is a lack of extensive research using modern forehead EEG monitors to analyze EEG patterns and processed EEG indices in isoflurane-alone-anesthetized dogs.

This study aimed to evaluate the feasibility of using EEG to track changes in the anesthetic level during isoflurane anesthesia in healthy canines. We sought to determine if EEG indices could accurately reflect variations in the anesthetic level and explore whether these indices were correlated with changes in cardiovascular parameters. By utilizing modern forehead EEG monitoring, we aimed to provide insights into the potential of real-time brain state monitoring for assessing the level of isoflurane-induced anesthesia and its relationship to cardiovascular function. We hypothesized that the use of frontal EEG recordings would be able to track brain state changes in real-time, correlating with changes in anesthetic level in dogs based on isoflurane MAC (minimum alveolar concentration) multiples.

## 2. Materials and Methods

### 2.1. Animals

Six healthy, male beagle dogs were utilized in this prospective study. All animals were 16 months of age and weighed between 10 and 13 kg. Prior to the study commencement, each beagle underwent a physical examination and blood work, including complete blood count, serum biochemistry, fecal, and urine analysis was conducted. On the evening preceding anesthesia, animals were fasted for eight hours with continued access to water. The sample size was determined based on a preliminary study of PSI (patient state index) and end-tidal isoflurane changes in different MAC multiples. Using a power analysis with a desired power of 80% and a significance level of 0.05, a sample size of 6 dogs was determined to be sufficient to detect significant differences between phases of isoflurane anesthesia. The Purdue University Animal Care and Use Committee approved the study protocol (#2108002178) and animal use procedures.

### 2.2. Experimental Design and Treatment Timeline

**Figure 1:**
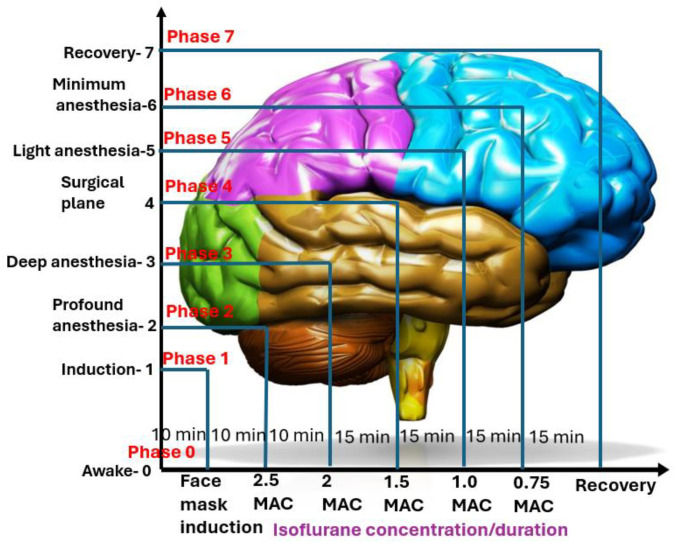
This schematic illustration depicts the treatment timeline for six dogs exposed to various end-tidal isoflurane concentrations. The x-axis represents the isoflurane concentration over time, while the y-axis indicates the different phases of treatment. Phase 0 (Awake—baseline) represents the dogs in their awake state, serving as the baseline. Phase 1 (Induction—face mask) involves induction with isoflurane using a face mask. Phase 2 (Profound anesthesia—2.5× MAC) involves maintaining isoflurane at 2.5× MAC for 10 min. Phase 3 (Deep anesthesia—2.0 MAC) involves reducing the concentration to 2.0× MAC and maintaining it for 15 min. Phase 4 (Surgical plane anesthesia—1.5× MAC) involves further reducing the concentration to 1.5× MAC for another 15 min. Phase 5 (Light anesthesia—1.0× MAC) involves decreasing the concentration to 1.0× MAC and maintaining it for 15 min. Phase 6 (Minimal anesthesia—0.75× MAC) involves lowering the concentration to 0.75× MAC for the final 15 min of maintenance. Phase 7 (Recovery) marks the recovery period, where the isoflurane concentration was terminated, allowing the dogs to regain consciousness. This figure provides a visual aid for the overview of the anesthesia treatment. The brain image suggests an approximate anatomical relationship for the phases, enhancing visual understanding despite not being precise.

Dogs were anesthetized with isoflurane at varying depths (levels) for 10–15 min each, from Phase 0 (awake) to Phase 7 (recovery) (Figure 1). The anesthetic level was measured in MAC multiples, with 1 MAC being an average of 1.3% end-tidal isoflurane based on previous studies [20,21]. EEG data were collected continuously, while cardiorespiratory vital signs, including electrocardiography, heart rate, respiratory rate, systolic, diastolic, and mean arterial blood pressure, hemoglobin saturation for oxygen with pulse oximetry, end-tidal CO_2_ (EtCO_2_), body temperature, and subjective anesthetic depth scores were collected every 3 min. However, both EEG and cardiorespiratory data were specifically recorded at the end of each phase. The details of the treatment timeline are listed as follows, as well as presented in Figure 1:

o **Phase 0**: Awake Baseline
  The treatment began by obtaining a short baseline measurement of the dog’s EEG and cardiorespiratory vital signs while the dog was fully awake. The procedures took approximately 10 min.
o **Phase 1**: Induction
  Isoflurane was administered via a face mask to induce anesthesia in the instrumented dog. During this phase, the dog transitioned from a conscious state to an unconscious state while EEG was monitored whenever possible. Once endotracheal intubation was completed, the dog was placed on a mechanical ventilator, marking the end of this phase. The procedure took 10 min.
o **Phase 2**: Profound anesthesia
  A deep level of anesthesia was initiated by deepening the anesthetic dose until the end-tidal isoflurane concentration reached 2.5× MAC (3.3% end-tidal). To mitigate the profound anesthetic effect, this phase was limited to 10 min of maintenance instead of the usual 15 min for the other phases.
o **Phase 3**: Deep anesthesia
  The level of anesthesia was reduced to a deep plane of 2× MAC (2.6% end-tidal) and maintained at this concentration for 15 min.
o **Phase 4**: Surgical plane
  The level of anesthesia was further reduced to a surgical plane, maintained using a concentration of 1.5 MAC (1.9% end-tidal) isoflurane. This phase also lasted for 15 min.
o **Phase 5**: Light anesthesia
  The level of anesthesia was further reduced to a light anesthesia of 1× MAC (1.3% end-tidal), maintained during this phase for 15 min.
o **Phase 6**: Minimal anesthesia
  The level of anesthesia was reduced to a minimal level of 0.75× MAC (0.9% end-tidal) isoflurane and maintained for 15 min or until the dog showed signs of readiness for extubation, such as coughing or gagging against the endotracheal tube.
o **Phase 7**: Recovery
  During this final phase of anesthesia, isoflurane administration was terminated. If the dog was ready for extubation, it was extubated and allowed to recover. All extubated dogs were monitored until they regained awareness. Recovery behavior was noted for any untoward signs of rough behaviors, such as thrashing, paddling, and vocalization, with or without salivation.

### 2.3. Face Mask Induction, EEG, Cardiorespiratory, Analgesic, and Behavioral Monitoring

#### 2.3.1. Isoflurane Face Mask Induction

Baseline EEG, HR, and non-invasive blood pressure (Phase 0) were recorded prior to induction. Face mask induction was initiated by gently restraining the dogs and exposing them to a 5% isoflurane concentration in a semi-closed circuit with a 4 L/min flow of 100% oxygen. Anesthetic depth was judged by the absence of toe pinch withdrawal, loss of righting reflex, relaxed jaw and muscle tone, sluggish palpebral reflex, and readiness for intubation.

Following endotracheal intubation, mechanical ventilation was initiated using a ventilator (Hallowell EMC 2002 ventilator, Pittsfield, MA, USA) and was set in volume-controlled mode. The EtCO_2_ was maintained between 35 and 45 mmHg by adjusting the tidal volume to 13–18 mL/kg, respiratory rate to 8–12 breaths per minute, and peak airway pressure to less than 15 cmH_2_O. These adjustments were made to ensure the EtCO_2_ remained within the target range of 35–45 mmHg. Cardiorespiratory parameters were recorded again at this point (Phase 1). Isoflurane concentration was subsequently increased to a target of 2.5× MAC, reaching a stable end-tidal concentration of 3.3% to achieve the start point of profound anesthesia. This concentration was then maintained for 10 min (Phase 2). During isoflurane anesthesia, a balanced electrolyte crystalloid fluid (Plasma-Lyte, Baxter International Inc. Deerfield, Illinois, USA) was administered at a rate of 10 mL/kg/hour via a preplaced intravenous catheter in one of the cephalic veins. This increased fluid rate of 10 mL/kg/h, compared to the usual 5 mL/kg/h, was justified to potentially counteract the profound vasodilation associated with isoflurane at higher MAC multiples.

#### 2.3.2. Electroencephalography Instrumentation

For EEG recording in dogs, six needle electrodes were subcutaneously placed. Electrode positions were adapted from the human 10–20 system, similar to our previous canine study [14]. Briefly, the R1 electrode was positioned at Fp2, and the L1 electrode was placed at Fp1. Additionally, the R2 electrode was midway between F4 and F8, while the L2 electrode was midway between F3 and F7. The ground electrode was located on the mid-sagittal central line, and the reference electrode was placed cranially on the midline. These electrodes were required to pass impedance checks using the SedLine^®^ monitor (Masimo Corporation, Irvine, CA, USA) to ensure proper functioning. The SedLine^®^ monitor displayed the EEG waveforms, patient state index (PSI) trend graph, and the density spectral array (DSA), with EEG indices to assess the dogs’ brain state in real time.

#### 2.3.3. Cardiorespiratory Monitoring

Physiological parameters, including SpO_2_, blood pressures (systolic, mean, and diastolic), EtCO_2_, and isoflurane, were recorded every three minutes throughout the study using a multiparameter monitor (Digicare LW9XVet, Lifewindow, Digicare Biomedical Technology, Boynton Beach, FL, USA). Electrocardiographic monitoring with Lead II configuration provided continuous assessment of cardiac rhythm and HR. Hemoglobin oxygen saturation was monitored using a pulse oximeter positioned on the tongue when feasible; otherwise, it was placed on the toe web. Indirect oscillometric blood pressure, including systolic, diastolic, and mean arterial pressure, was obtained using an appropriately sized blood pressure cuff (cuff width was 40% of the limb circumference). The respiratory rate, end-tidal carbon dioxide, and anesthetic concentration were continuously monitored via a side-stream capnography. Rectal temperature was also recorded. Blood pressures (systolic, mean, and diastolic) and HR measurements, in addition to subjective anesthetic depth scores, were used as part of an assessment tool for anesthetic depth (Appendix A).

#### 2.3.4. Nociceptive Assessment

To evaluate antinociceptive effects, transcutaneous electrical nerve stimulation was administered in a manner similar to a previous study [14]. Two 25-gauge needles were inserted into the skin over the lateral aspect of the tibia, spaced five centimeters apart. A nerve stimulator (SunStim Nerve Stimulator, Ministim, Tri-Animal Health Services, Dublin, OH, USA) delivered a 0.22 ms square-wave pulse stimulus at 400 V with a frequency ranging from 0 to 100 Hz. The stimulation duration was three seconds. The intensity of the stimulus was adjusted until the animal ceased to exhibit any purposeful movement in response to the stimulation, such as swallowing, head shaking, or limb movement. Nociceptive assessment was performed after the collection of physiological data at three-minute intervals.

#### 2.3.5. Behavioral Assessment of the Anesthetic Level

Behavioral assessment (Table A1) was conducted every three minutes to evaluate anesthetic level, concurrent with physiological parameter monitoring. Auditory responsiveness was assessed using a clicker. A positive response was defined as the head turning towards the sound source or ear twitching. This assessment was performed both prior to anesthetic induction, during maintenance, and recovery. Additionally, during the maintenance phases, a subjective depth score was assigned to the subjects every 3 min (Appendix A). The subjective depth score was adapted from one of our previous studies with propofol in dogs [15]. Purposeful movements such as limb withdrawal, head or neck motion, tail or nose twitching, swallowing, or coughing in response to stimulation were considered positive behavioral responses. Jaw tone was evaluated through manual manipulation, while palpebral and corneal reflexes were assessed by eliciting a response or lack thereof to saline drops (0.9% Sodium Chloride Injection, USP, Baxter International Inc. Deerfield, Illinois, USA) applied to the eyelids and cornea, respectively. For assessing the quality of recovery, a straightforward categorization system of smooth, acceptable, and rough recovery was used. The categories were defined as follows: smooth recovery (uneventful), acceptable recovery (verbal assurance needed to ensure calmness), and rough recovery (physical restraint required to prevent self-injury).

### 2.4. EEG Data Acquisition, Analysis, and Correlation with Cardiovascular Parameters

Processed EEG data, including PSI, burst suppression ratio (SR), electromyography (EMG) activity, 95% spectral edge frequency (SEF95), and artifact (ART) activity, were continuously collected and stored as CSV files by the SedLine monitor. Raw EEG data were also obtained for visual examination with EDF files. More than 20,000 data points per animal were generated for specific variables. As previously described [14], the SedLine monitor utilizes forehead electrodes to quantify facial muscle activity, which is incorporated into the calculation of the PSI, a marker of anesthesia and relaxation. To overcome technical challenges, raw EEG data underwent resampling to 89 Hz before constructing the DSA using MATLAB’s (2024b, MathWorks, Natic, MA, USA) pwelch function [22].

Statistical comparisons of mean values for PSI, SR, SEF95, EMG, and ART across experimental phases were conducted using linear mixed models with repeated measures (SAS 9.4, SAS Institute Inc., Gary, NC, USA). Statistical significance was determined at a *p*-value < 0.05, with post-hoc comparisons and Bonferroni correction applied as needed. Results are presented as mean ± standard deviation for both hemodynamic and EEG parameters in each phase. To explore the association between the EEG index PSI and cardiovascular parameters (HR and mean arterial blood pressure—MBP) across varying anesthetic depths, Spearman’s rank correlation coefficient (ρ) tests were performed.

## 3. Results

### 3.1. EEG Indices Changes with Different Isoflurane Anesthetic Concentrations (Phase 0–7)

Table 1 presents the evolution of EEG indices in dogs as they progressed from an awake baseline to induction, maintenance at various levels of isoflurane anesthesia, and subsequent recovery. The statistical analysis revealed significant differences (*p* < 0.01) in blood pressure (systolic, mean, and diastolic), and EEG indices across all treatment phases.

**Figure 2:**
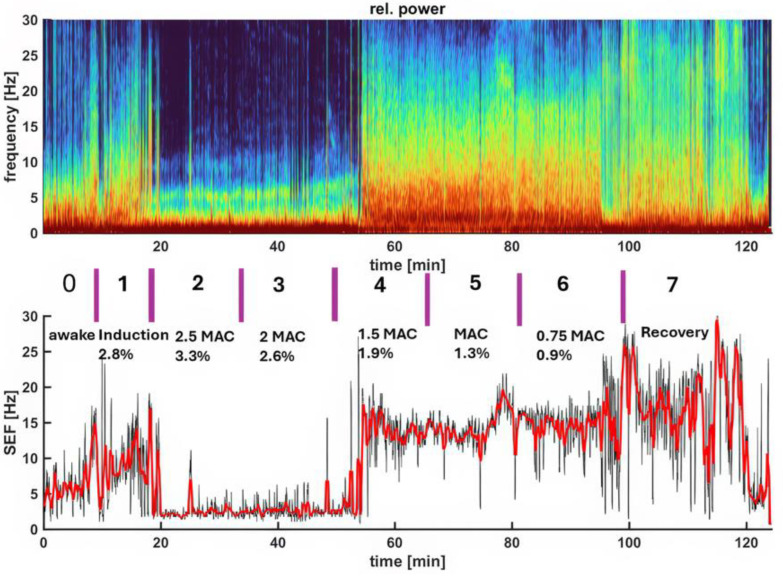
The spectrogram (upper panel) of a study dog illustrates the evolution of EEG patterns from awake (Phase 0) to isoflurane induction (Phase 1) and through various levels of isoflurane anesthesia (Phase 2–6), as indicated by MAC multiples and end-tidal isoflurane concentrations, to recovery (Phase 7). The 95% spectral edge frequency of the combined left and right hemispheres is depicted in the lower panel. Numbers separated by the purple lines represent the study phases (0–7). Burst suppression, characterized by dark blue regions in the upper panel and EEG frequency in the lower panel, was clearly evident during deeper anesthesia (2.5 and 2 MAC). As the levels of anesthesia lightened (1.5×, 1×, 0.75× MAC, and recovery), a distinct shift in SEF95 frequencies was observed, transitioning from burst suppression and delta dominance to alpha- and beta-band activity. As the dog recovered further, frequencies entered the high beta- and gamma-band activity.

**Figure 3:**
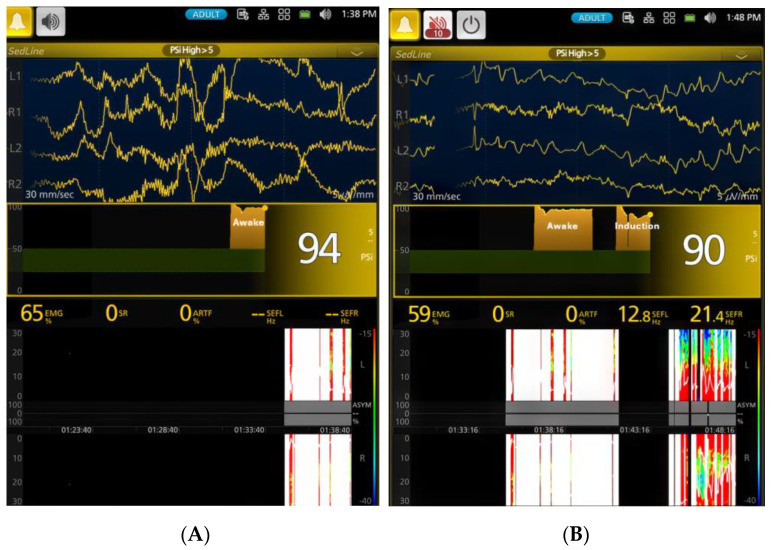
Panel (**A**) (left—timestamp 1:38 p.m.) shows an awake EEG pattern in the dog (Phase 0), characterized by high-frequency gamma waves, low amplitudes, and a high PSI (94) value. Due to high muscle activity (EMG 65%), the white color on the SedLine monitor’s spectrogram typically indicates periods of artifact that may be caused by such muscle activity, preventing the display of SEF95 values. Panel (**B**) (right—timestamp 1:48 p.m.) depicts the EEG pattern after isoflurane face mask induction and intubation (Phase 1), transitioning to profound anesthesia. The PSI trend graph, located in the middle of the figures, provides a visual representation of the PSI value changes over time, allowing clinicians to monitor changes in the patient’s level of consciousness and anesthetic depth. The PSI trend graphs in both panels (**A**,**B**) show high PSI values (the yellow blocks). The high-frequency awake waveform disappeared, transitioning to the typical isoflurane EEG pattern of alpha (SEF95 12.8 Hz) and beta (SEF95 21.4 Hz) waveforms. As the dog was in the early stages of transition, muscle activities remained high (EMG 59%). The spectrogram at the bottom of the figures transitioned from a pattern of high muscle activity and artifacts (Panel A - white color with some red color) to a typical light anesthesia pattern (Panel B) characterized by a mixture of high alpha and beta frequency colors (red-green and blue) and a reduction in muscle activity.

**Figure 4:**
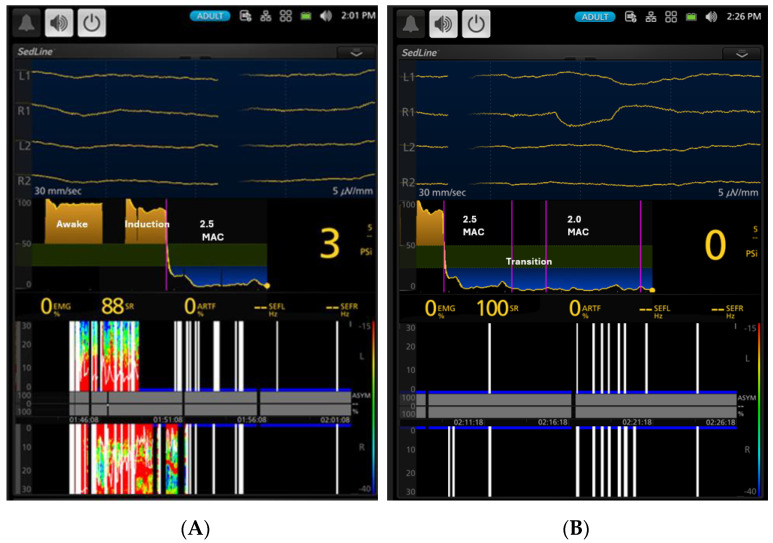
Panel (**A**) (left figure) illustrates a state of EEG electrical silence in the dog after 10 min of exposure to 2.5× MAC (3.3% end-tidal) isoflurane. The dog exhibited a burst suppression ratio of 88%, and the PSI value was 3. The PSI trend graphs demonstrate a profound depth of anesthesia, indicated by the deep blue color during both 2.5× (**A**) and 2× MAC (**B**), which falls well below the default unconscious range of 25–50 PSI (the green zone in the PSI trend graph figure between 25 and 50 PSI). The spectrogram showed bilateral burst suppression with electrical silence, characterized by the presence of black color blocks with blue tip lines in both hemispheres. Panel (**B**) (right, timestamp 2:26 p.m.) depicts a state of total electrical silence with 100% burst suppression and a PSI value of zero at 15 min after anesthesia with isoflurane concentration of 2× MAC (2.6% end-tidal). The vertical pink lines demarcate the duration of 2.5× MAC, the transition from 2.5× MAC to 2× MAC, and the subsequent maintenance phase at 2× MAC. The white bars indicate periods of signal loss or poor quality, likely due to significant electrical silence and the profound burst suppression. The spectrogram at the bottom of the figures transitioned from a pattern of light plane of anesthesia characterized by red-green and blue colors (Panel **A**) to a high percentage of burst suppression (black color) (Panel **B**). The white line indicates artifacts during the EEG quiescence.

**Figure 5:**
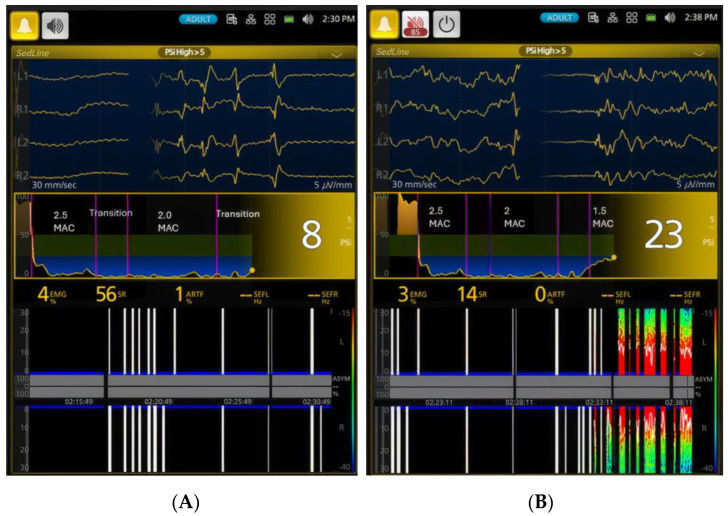
This figure illustrates two types of burst suppression patterns. The burst suppression EEG pattern is characterized by alternating periods of high-voltage electrical activity waves (bursts) and periods of electrical inactivity or flat lines (suppression) in the brain. Panel (**A**) illustrates a type of burst suppression EEG waveform, characterized by bursting activity in the middle of the screen and long silent waveforms on either side. These waveforms were observed following exposure to a profound depth of anesthesia with prolonged isoelectric periods. In this case, electrical silence occurred during the profound and deep anesthesia of 2.5× and 2× MAC, and the dog was emerging from these periods. Panel (**B**) shows a different burst suppression pattern that occurred as the dog was in the surgical plane of anesthesia (1.5× MAC), when the isoflurane was not as profound as in 2.5× and 2× MAC but still profound enough to induce a burst suppression pattern. This burst suppression pattern was characterized by a short silent wave in the middle, while mixed with alpha- and beta-band activity and forming a distinct spectrogram that is easily discernible when comparing the spectrograms of (**A**). In (**A**), the spectrogram power remains low (all black color) due to electrical silence with some burst suppression (56%), whereas in (**B**), the burst suppression percentage reduces significantly to 14%, and other EEG power increases as indicated by the presence of red and green colors, suggesting the awakening of various brain regions from a previously profoundly depressed phase.

**Figure 6:**
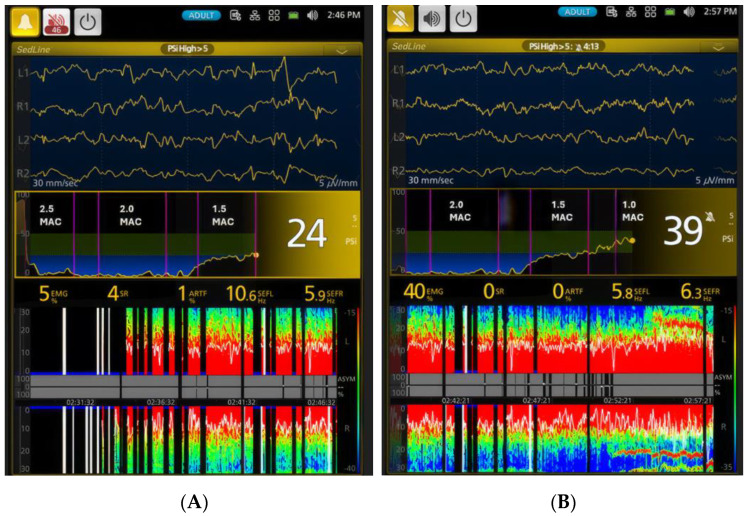
Panel (**A**) illustrates the unique EEG pattern of the isoflurane surgical plane of anesthesia (Phase 4, 1.5× MAC) in dogs. The waveforms were dominated by alpha and beta activity (see Table 1 SEF95 values). The PSI was 24 with low SR of 4% and EMG of 5%. Panel (**B**) shows that as the depth of anesthesia lightened to 1× MAC, the PSI went up to 39, and EMG activity increased to 40%, visible on the spectrogram as a red line pattern spreading over time at high frequencies in the gamma-band range. The PSI trend graphs also provided a clear indication that the isoflurane anesthesia level went from profound depression to a much lighter plane of anesthesia over time. The spectrogram at the bottom of the figures shows the typical surgical plane of EEG powers, characterized by alpha and beta activity represented by red and green color blocks in both Panel (**A**) and Panel (**B**). Within the red block spectrogram, the 95% SEF, represented by the white lines, appears to fluctuate up and down.

**Figure 7:**
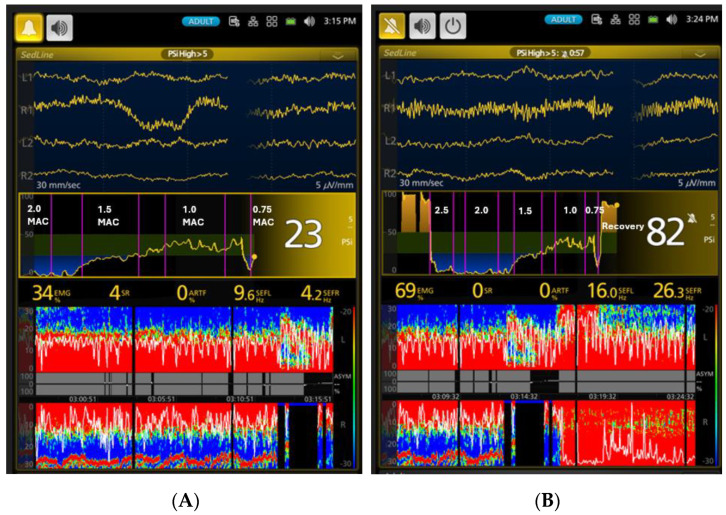
(**A**) The general PSI trend graph shows an upward swing from 1.5× MAC to 0.75× MAC over time, with fluctuations and a sudden dip due to burst suppression (see the black block in the figure). (**B**) When the anesthetic concentration was reduced from 1× MAC to 0.75× MAC, there was a noticeable increase in EMG activity in both the spectrogram (as evidenced by the red-colored line spreading over time in the bottom part of the spectrogram) and the raw EEG waveforms. The figure clearly depicts continuous brain state changes leading to recovery, shown by increasing muscle activity and a significant rise in PSI values. SEF95 values also increased to beta-band (16.0 Hz) and low gamma-band (26.3 Hz) frequencies, indicating regained consciousness. Additionally, significant changes in the red areas of the spectrogram indicate higher frequency and lower amplitude of EEG power and muscle activity, suggesting greater awareness. The raw EEG, EEG indices, and spectrogram in both (**A**,**B**) collectively provided valuable clues to indicate real-time changes in anesthesia depth, guiding clinicians in managing isoflurane anesthesia.

Figure 2 depicts a spectrogram and the SEF95 trend, illustrating EEG changes in a representative dog during the awake state, isoflurane induction, various levels of isoflurane anesthesia, and the recovery period. The most distinct isoflurane EEG patterns, identifiable in all dogs, including this representative one, are the EEG wave depression during the profound and deep anesthesia (2.5× and 2× MAC) period and the dominance of beta-band and alpha-band activity during the surgical plane (1.5× MAC) and light anesthesia (1× MAC) periods. Figure 3, Figure 4, Figure 5, Figure 6 and Figure 7 display screenshots of EEG waveforms, EEG indices, PSI trend graphs, and spectrograms during the various anesthetic depths of the same representative dog. These figures clearly demonstrate the transition in EEG patterns and EEG indices from induction to deep anesthesia, followed by a transition from deep to light plane anesthesia, and ultimately to recovery.

Anesthetic induction, endotracheal intubation, and instrumentation were achieved within 10 min for all dogs in this study. Compared to the awake state, induction resulted in a significant (*p* < 0.01) decrease in mean arterial blood pressure from 108.5 ± 16.7 mmHg to 60.0 ± 4.4 mmHg, indicating entry into a plane of anesthesia characterized by low SR% and alpha- and beta-range SEF95 values. Concurrently, a significant (*p* < 0.01) decreased EMG activity suggested reduced muscle tone. Screenshots illustrating the awake EEG (Figure 3A) and the EEG waveforms soon after endotracheal intubation (Figure 3B) are provided.

As anesthesia deepened to 2.5× MAC, dogs entered the deepest anesthetic plane (Figure 4), as evidenced by a markedly significant (*p* < 0.01) decrease in PSI to 6.5 ± 10.8, an increase in SR% to 78.3 ± 23.9%, and alpha-range SEF95. The PSI trend graph (ranging from 0 for deep anesthesia to 100 for fully awake over time) indicates that the dogs were in a profoundly deep level of anesthesia during these periods, with PSI values (Figure 4A,B) well below the default unconscious range of 25–50 psi. This default PSI range is based on empirical algorithms that analyze EEG frequency bands, phase information from anteroposterior synchronization, and bilateral coherence of the brain. Muscle tone significantly (*p* < 0.01) decreased from 52.7 ± 33.5 to 7.4 ± 11.6, indicating profound relaxation. At 2× MAC, dogs remained deeply anesthetized with similarly low PSI and high SR%, and muscle tone remained minimal.

The raw EEG waveforms exhibited two distinct burst suppression patterns. One pattern (Figure 5A) occurred during the transition into and immediately out of the profound anesthesia-induced isoelectric-dominant phases (2.5× and 2× MAC). The other pattern (Figure 5B) appeared after the dog emerged from the isoelectric silence phases and transitioned into the surgical plane of anesthesia (1.5× MAC). The first pattern featured a longer silence period followed by a shorter burst of activity. Conversely, the second pattern had a shorter silence period followed by a longer burst of activity. The first burst suppression waveform pattern may indicate a profound stage of brain depression (Figure 5A). The second pattern represents a burst suppression pattern emerging from profound brain depression, mixed with alpha- and beta-band EEG activity, creating a unique spectrogram pattern (Figure 5B). This may suggest the activation of different brain regions following a period of profound depression with isoelectric silence.

Transitioning to the surgical plane of anesthesia (1.5× MAC), PSI significantly increased to 31.8 ± 12.4, while SR% markedly decreased to 9.6 ± 20.6% from the previous phase. In this phase, Phase (5), SEF95 values were predominantly in the alpha and low beta range (Table 1), with minimal delta waves and occasional burst suppression waveforms (Figure 6A). At the lighter plane of anesthesia (1× MAC), PSI increased further, SR% remained minimal, SEF values stayed within the alpha–beta range, and moderate muscle tone was evident. The PSI trend graphs in Figure 6A,B clearly demonstrate how isoflurane anesthesia depth change over time with varying isoflurane MAC multiples. During both the surgical plane and light anesthesia, EEG patterns were characterized by a predominance of alpha- and beta-band activity.

As anesthesia lightened to 0.75× MAC, PSI and SR% returned to near-awake levels, and muscle tone increased significantly (*p* < 0.01). Interestingly, occasional burst suppression occurred (Figure 7A), suggesting the redistribution of isoflurane within the body’s various compartments and its non-simultaneous elimination, possibly in waves or bursts rather than at a constant rate. Only two dogs completed this phase; the remaining four showed signs of readiness for extubation and were subsequently extubated and entered the recovery phase. Finally, during recovery, all dogs exhibited high PSI, low SR%, and an EEG pattern similar to awake waveforms (Figure 7B), accompanied by strong muscle activity, indicating a return to wakefulness.

### 3.2. Cardiovascular Parameters, Tolerance to Electrical Stimulation, and Subjective Anesthetic Depth Scores of the Dogs during the Various Depths of Isoflurane Anesthesia

Table 2 presents cardiovascular parameters, tolerance to electrical stimulation, and subjective anesthetic depth scores across different isoflurane concentrations.

During the awake baseline (Phase 0), HR was 97.1 ± 20.8 bpm, and blood pressure was high with a mean arterial blood pressure (MBP) of 108.5 ± 16.7 mmHg. No electrical stimulation was performed at this time. After endotracheal intubation (Phase 1), the end-tidal isoflurane concentration increased to 2.8 ± 1.3%, HR increased (119.5 ± 26.2 bpm), but MBP decreased (60 ± 4.4 mmHg) significantly. A subjective depth score of 3.0 ± 0.0 indicated light anesthesia. Electrical stimulation was not performed at this time.

At 2.5× MAC (Phase 2), HR remained stable (121.4 ± 13.6 bpm), while blood pressure decreased significantly (*p* < 0.001) (MBP 45.6 ± 16.4 mmHg). A high tolerance to electrical stimulation (888 ± 33.2 Hz) and a depth score of 2.1 ± 0.7 indicated profound anesthesia, corresponding to an end-tidal isoflurane concentration of 3.3 ± 0.1%.

At 2× MAC (Phase 3), HR was 120.9 ± 12.4 bpm with a slight return of MBP (50.4 ± 7.4 mmHg) due to reduced isoflurane (2.6 ± 0.1%). The electrical stimulation tolerance and the depth score remained similar to Phase 2.

As the dog entered the surgical plane of anesthesia (1.5× MAC, Phase 4), maintaining an end-tidal isoflurane concentration of 1.9 ± 0.1%, heart rate (HR) and mean blood pressure (MBP) increased significantly (*p* < 0.001). However, electrical stimulation tolerance and the depth score remained consistent with previous phases, suggesting that these two parameters may not accurately reflect anesthetic depth.

Reducing the isoflurane concentration to 1× MAC (Phase 5) resulted in a significant increase in both HR and MBP (*p* < 0.001). Electrical stimulation tolerance decreased, and the depth score increased, indicating a lighter anesthetic plane.

Finally, at the minimum anesthesia level of 0.75× MAC (Phase 6), HR remained stable while MBP continued to rise. Due to the minimal anesthetic depth, electrical stimulation was not performed as it caused the dog to become aroused and extubated. Despite the lack of stimulation, four dogs were spontaneously extubated during this phase. A depth score of 4 ± 0 was assigned.

During recovery (Phase 7), HR and blood pressures increased significantly (*p* < 0.001) compared to baseline (HR 153.6 ± 19.3 bpm, MBP 122.9 ± 25.9 mmHg). No electrical stimulation was performed, and a depth score of 4.7 ± 0.5 indicated the recovery of consciousness.

During the study, temperatures were maintained within a range of 99.7–101.5 °F, respiratory rates within 13–24 bpm, EtCO_2_ levels were maintained within 34–44 mmHg, and pulse oximetry within 96–98% in all the dogs.

To examine the relationships between PSI, HR, and MBP, Spearman’s rank correlation coefficients were calculated. The results showed a weak negative correlation between PSI and HR (ρ = −0.2089, *p* = 0.2497), indicating no significant association. Conversely, a strong positive correlation was observed between PSI and MBP (ρ = 0.8098, *p* = 0.001), suggesting a reliable relationship. The correlation between HR and MBP was very weak and non-significant (ρ = 0.0733, *p* = 0.6885).

The recovery times from extubation to gaining sternal recovery with normal behaviors for the six dogs ranged from 6 min and 14 s to 12 min and 26 s. The median recovery time was 9 min and 14 s. Five of the six dogs received a rough recovery score (from the three categories: smooth, acceptable, and rough). The rough recovery occurred within 1–2 min of extubation and was characterized by vocalizations, paddling, and agitated behavior resembling delirium. Despite verbal reassurance from the handlers, these dogs exhibited a period of non-responsiveness to verbal commands and required physical restraint to prevent excessive physical activity. The duration of these behaviors varied among the dogs, but all the dogs settled down within 3–4 min. No further consequences were observed in these dogs after complete recovery.

## 4. Discussion

Several key findings can be identified from this study. First, it is possible to use frontal EEG to monitor the level of isoflurane anesthesia in real-time in dogs. This is similar to studies that have established a strong correlation between EEG changes and various anesthetic agents, including isoflurane, induced general anesthetic effects in humans [5,6,23,24]. Second, the current study also characterized the EEG pattern changes induced by isoflurane in dogs. For example, isoelectric mixed with burst suppression were the main EEG patterns at profound and deep isoflurane anesthesia. As the level of anesthesia reduced to the surgical plane of anesthesia, the EEG pattern changed to alpha- and beta-dominant waveforms. When anesthesia lightened further, the EEG activity changed to a typical awake EEG pattern characterized by rapid and small amplitude waveforms.

Thirdly, the current study demonstrated that dogs subjected to deep isoflurane anesthesia (2.5× and 2× MAC) exhibited unconsciousness and unresponsiveness. Their EEG patterns were characterized by predominantly isoelectric activity with occasional burst suppression. PSI values were very low, while SR% was high, EMG values were low, and the dogs were unresponsive to maximum electrical stimuli.

Isoflurane exerts its effects by interacting with various neurotransmitter receptors and ion channels within the central nervous system. These interactions result in the four primary anesthetic effects: unconsciousness, amnesia, muscle relaxation, and pain relief.

At the receptor level, isoflurane, like propofol, enhances the activity of gamma-aminobutyric acid (GABA) at the GABA_A receptors, which are inhibitory neurotransmitter receptors. This enhancement facilitates chloride ion influx, leading to neuronal hyperpolarization and reduced excitability. This mechanism is pivotal in inducing unconsciousness and amnesia as it diminishes overall brain activity, hindering memory formation and disrupting neural connectivity [5,6,23,25]. This disruption impairs the brain’s ability to integrate information, resulting in a reduction in consciousness [23,24]. Consequently, EEG patterns exhibit a predominance of high-amplitude, low-frequency delta, theta, and alpha waves.

While unconsciousness and unresponsiveness are often used interchangeably, they are not identical [26]. Anesthetics, such as isoflurane, reduce responsiveness by acting on endogenous sleep pathways, particularly by suppressing histamine release from the tuberomammillary nucleus [26,27]. This suppression affects subcortical structures such as the amygdala and basal ganglia, which are involved in learning, memory, motivation, emotion, and motor output [26,28,29]. The amygdala’s activity and connectivity are highly susceptible to anesthetics, leading to impaired decision-making and reduced responsiveness to stimuli. The putamen, part of the basal ganglia circuit controlling motor output, also shows reduced activity and connectivity under anesthesia, contributing to unresponsiveness by disrupting action selection [26,27,28,29,30,31]. Additionally, anesthetics like isoflurane affect the brainstem and ventral horn of the spinal cord, reducing motor tone and inhibiting reflex motor responses [26].

Beyond its effects on the brain’s higher-order functions, isoflurane also potentiates the inhibitory action of glycine at glycine receptors, particularly in the spinal cord. This potentiation significantly contributes to muscle relaxation and antinociception [32,33]. By augmenting glycine’s inhibitory effects, isoflurane facilitates muscle relaxation and reduces pain perception, which are crucial during surgical procedures. As observed in this study, these dogs exhibited unresponsiveness to electrical stimulation.

Furthermore, isoflurane modulates voltage-gated potassium and sodium channels, essential for action potential generation and propagation [32,33]. By stabilizing these channels in their inactive states, isoflurane further reduces neuronal excitability, contributing to the overall anesthetic effect. This modulation aids in maintaining the patient’s unconscious state and supports muscle relaxation by preventing excessive neuronal firing. This mechanism may explain the observed burst suppression and isoelectric EEG patterns during the profound and deep anesthesia observed at 2.5 and 2 times the minimum alveolar concentration (MAC) in this study.

In addition to its effects on inhibitory neurotransmission and ion channels, isoflurane inhibits N-methyl-D-aspartate (NMDA) receptors, which are involved in excitatory neurotransmission and synaptic plasticity [32,33]. This inhibition reduces excitatory neurotransmission, contributing to unconsciousness and amnesia. Additionally, isoflurane affects AMPA receptors, also involved in excitatory neurotransmission, further enhancing its anesthetic properties. By inhibiting these receptors, isoflurane ensures the patient remains unaware and unable to recall the surgical experience. This mechanism may explain the observed alpha with low beta EEG waves during isoflurane anesthesia, suggesting the inhibition or partial inhibition of NMDA receptors.

Overall, isoflurane induces its anesthetic effects by synergistically enhancing inhibitory neurotransmission, modulating ion channel activity, and inhibiting excitatory neurotransmission. These interactions result in a profound depth of anesthesia characterized by unconsciousness, muscle relaxation, amnesia, and antinociception, as reflected in the distinctive EEG patterns observed during various anesthetic depths. The dogs in the current study were totally unresponsive to auditory and electrical stimuli at 2.5× and 2× MAC concentrations. Moreover, the isoelectric brain waves suggest minimal communication between the cerebral cortex and subcortical structures, providing strong evidence that they were indeed unconscious and unresponsive during these profound levels of anesthesia [26,27,28,29,30,31,32,33].

Previous research has documented a paradoxical increase in the BIS index (bispectral index) in dogs [34,35] and cats [36] when exposed to elevated isoflurane concentrations. A recent study in mice, employing transdermal needle electrodes and a BIS monitor, also reported a similar phenomenon while monitoring varying isoflurane concentrations in mice [37]. These authors attributed this paradoxical increase in the BIS index at higher isoflurane levels (2.2%) to the predominance of respiratory signals during prolonged periods of suppression at these concentrations, potentially leading to the misleading nature of the BIS index [37]. In contrast to these studies, we did not observe a paradoxical increase in PSI values during the high isoflurane concentrations (3.2% and 2.6% vs 1.9% and 1.3%). This discrepancy may be attributed to the use of different EEG monitors, with different algorithms [38] and techniques among ours and their studies.

In the current study, there appeared to be a trend between the MAC multiples, end-tidal isoflurane concentration, HR, blood pressure, tolerance to electrical stimulation, and subjective depth assessment. As the MAC multiple increased, the end-tidal isoflurane concentration also rose, leading to observable changes in physiological parameters. At higher MAC multiples (2.5× and 2×), HR remained relatively stable, but blood pressure (SBP, MBP, and DBP) decreased significantly, indicating a deeper anesthetic state. Tolerance to electrical stimulation was high at these levels, with values close to the maximum of 900 Hz, and the depth score indicated a deep level of anesthesia (scores around 2.1 to 2.3). As the MAC multiple decreased, the end-tidal isoflurane concentration dropped, HR increased, and blood pressure rose, reflecting a lighter anesthetic state. The tolerance to electrical stimulation decreased significantly at 1× MAC, and the depth score increased to around 3.7, indicating a lighter anesthesia. During recovery, the end-tidal isoflurane concentration was minimal, HR and blood pressure were elevated, and the depth score approached 4.7, indicating near wakefulness. These findings corroborated our clinical observations that higher MAC multiples were associated with significant physiological depression and unresponsiveness to nociceptive stimuli in dogs anesthetized with isoflurane [39]. This suggests that higher MAC multiples indeed induce a state of profound depressive brain activity besides the cardiovascular functions. The observed decrease in blood pressure with increased isoflurane MAC multiples was primarily due to vasodilation and reduced myocardial contractility, while the stable HR was maintained through compensatory mechanisms such as the baroreceptor reflex and increased sympathetic nervous system activity [40,41].

The PSI is a sophisticated and complex tool designed to assess the impact of anesthesia and sedation on patients [42]. Utilizing a high-resolution four-channel EEG such as the SedLine monitor used in the current study, the PSI employs a refined algorithm to process data, meticulously filtering out artifacts. This index is particularly valuable in operating rooms, providing real-time insights into a patient’s level of anesthesia and their response to anesthetics in humans [42]. Our PSI data support this notion and its applicability to isoflurane-anesthetized dogs.

The analysis using Spearman’s rho (ρ) correlation coefficient revealed that the EEG index PSI has a much stronger correlation with MBP than with HR. Specifically, there is a positive correlation between PSI and MBP (ρ = 0.8098, *p* = 0.001), indicating a consistent and closely linked relationship across different anesthetic depths. In contrast, the correlation between PSI and HR is weak (ρ = −0.2089, *p* = 0.2497), showing no significant association. The correlation between HR and MBP was very weak and non-significant (ρ = 0.0733, *p* = 0.6885). This suggests that MBP is a more reliable indicator of changes in PSI compared to HR during anesthesia. A similar significant correlation between PSI and arterial blood pressure during anesthesia has been reported in propofol-anesthetized humans [42,43,44] and dogs [15].

Different anesthetics or sedatives produce distinct EEG patterns and signatures, likely linked to their interaction with different receptor sites located in various regions of the brain. For example, propofol induces distinct alpha and delta waves, while ketamine is known for its unique dissociative effects by blocking NMDA (N-methyl-D-aspartate receptors) in the brain and spinal cord, often resulting in high-frequency gamma oscillations [6,7,8,10]. Both propofol and isoflurane share a dominant mechanism of action by enhancing GABAergic inhibition to induce general anesthesia [6,7,8,10]. At the surgical plane of anesthesia, propofol typically produces a pattern of high-amplitude slow waves and alpha oscillations, while volatile anesthetics like isoflurane generate a similar pattern but also induce theta oscillations. When the anesthetic concentration deepens, both propofol and isoflurane induce slow waves and burst suppression at deeper levels [6,7,8,10].

In the current study, when isoflurane was at 1–1.5× MAC as at the surgical or light plane of anesthesia, we observed minimal theta waves but mostly alpha and low beta oscillations in the dogs. However, in the deeper plane of anesthesia (2–2.5× MAC), the burst suppression pattern appeared with an isoelectric pattern and minimal delta waves. This is quite different from our previous study of propofol in dogs [14], where we identified delta oscillations and burst suppressions, but minimal alpha and rarely any beta waves. These differences between the current study and the dogs anesthetized with propofol and humans with isoflurane and propofol were likely due to either the drug mechanism or species variation differences.

The high incidence of dysphoria and delirium-like rough recoveries in five of the six dogs in this study, particularly given the deep levels of anesthesia achieved with isoflurane, exposes evidence that overly anesthetizing dogs may likely result in such sequelae. The proposed reasons for these dysphoric recoveries include prolonged deep anesthesia, neurotransmitter imbalance, GABAergic and glutamatergic system dysregulation, and activation of the hypothalamic–pituitary–adrenal (HPA) axis, resulting in high levels of stress. Additionally, hypotension may have played a role in causing the imbalance of these neuromechanisms. Since we did not measure the cerebral oxygenation, despite the SpO_2_ being within acceptable levels in these dogs, it is still possible that the profound and prolonged hypotension played a role due to ischemic stress to the brain.

The EEG patterns observed in these dogs, which mainly showed high beta-band and gamma-band activity with muscle artifacts, reflect an excitatory nature that could be indicative of a recovering ischemic brain. However, it is not uncommon in clinical situations involving dogs anesthetized with isoflurane and experiencing hypotension and over-anesthesia to observe such rough recoveries. In human studies [45,46,47,48], high burst suppression ratios and isoelectric activity have been implicated in postoperative delirium, especially in geriatric or neurologically compromised patients, such as those with dementia. While it is uncertain whether the same mechanisms apply to dogs, the unusually high percentage of dogs in this study experiencing hypotension and high suppression ratios suggests that these factors may contribute to rough recoveries.

These findings emphasize the clinical importance of using multimodal, balanced anesthetic protocols to lower volatile anesthetic concentrations while maintaining adequate anesthesia. By combining isoflurane with various other agents, such as opioids, sedatives, dissociative anesthetics, local anesthetics, gabapentinoids, and even cannabinoids, clinicians may be able to reduce the incidence of postoperative neurocognitive complications and improve overall patient outcomes.

The present study has several limitations. The use of male beagle dogs may limit the generalizability of the findings to other dog breeds and sexes. Additional non-invasive monitoring techniques, such as perfusion and plethysmographic variability indices [49], could have been incorporated to enhance cardiorespiratory assessment. Moreover, the measurement of SpHb by co-oximetry and invasive blood pressure monitoring may have provided more comprehensive hemodynamic data. The absence of a study group using BIS as a comparison to SedLine is another limitation. Finally, the awake EEG measurements, including those obtained during face mask induction and recovery, were influenced by individual canine temperament and muscle activity artifacts, which can affect EEG signal quality.

## 5. Conclusions

In conclusion, this study demonstrates the utility of EEG monitoring to track isoflurane anesthesia at various levels in dogs in real-time. The findings reveal a strong correlation between PSI and mean arterial blood pressure (MBP), and a weak correlation between PSI and heart rate (HR), highlighting MBP as a more reliable indicator of anesthetic depth. Unique EEG characteristics of isoflurane were identified, with alpha- and low beta-band oscillations predominating at the surgical plane of anesthesia, and predominantly isoelectric patterns with burst suppression observed at profound deep anesthesia levels. These insights underscore the importance of EEG as a tool for assessing anesthetic depth and ensuring optimal anesthesia management. Clinical utilization of EEG and MBP can help avoid excessive isoflurane maintenance, thereby reducing the risk of rough recovery. Further research is needed to identify the EEG patterns resulting from the combination of signatures of individual anesthetics, such as dexmedetomidine, propofol, ketamine, and inhalant anesthetics in dogs, to enhance our understanding and improve clinical outcomes.

## Figures and Tables

**Table 1:** EEG indices of six dogs subjected to isoflurane were recorded from awake baseline (Phase 0) to induction (Phase 1), and then to profound anesthesia (Phase 2, 2.5× MAC-minimum alveolar concentration), deep anesthesia (Phase 3, 2× MAC), surgical plane (Phase 4, 1.5× MAC), light anesthesia (Phase 5, 1× MAC), minimum anesthesia (Phase 6, 0.75× MAC), and recovery (Phase 7). The data for the patient state index (PSI), burst suppression ratio (SR), electromyography (EMG) activity, 95% spectral edge frequency (SEF95) for the left (SEF-L) and right (SEF-R) hemispheres, and artifact (ART) activity are presented as mean ± standard deviation. The * indicates that there is a significant difference (*p* < 0.01) between each treatment phase. Within the column, different letters (a, b, and c) indicate significant differences. The same letter indicates no significant difference.

Phases	PSI *	SR% *	SEF-L	SEF-R	EMG *	ART *
Baseline (0)	72.8 ± 29.1 a	5.3 ± 6.5 a	9.3 ± 7.6	6.9 ± 6.1	52.7 ± 33.5 a	12.5 ± 18.1 a
Induction (1)	65.0 ± 22.5 a	3.9 ± 5.1 a	7.7 ± 4.8	7.3 ± 6.3	27.6 ± 27.0 b	21.6 ± 26.3 a
2.5× MAC (2)	6.5 ± 10.8 b	78.3 ± 24.0 b	10.8 ± 4.3	8.3 ± 5.3	7.4 ± 11.6 c	5.2 ± 12.4 b
2× MAC (3)	10.5 ± 13.6 b	63.0 ± 33.3 b	15.2 ± 7.8	15.9 ± 7.5	6.4 ± 12.9 c	17.2 ± 35.4 a
1.5× MAC (4)	31.8 ± 12.4 c	9.6 ± 20.6 a	13.5 ± 4.0	11.4 ± 4.3	3.9 ± 4.4 c	2.2 ± 4.8 b
1× MAC (5)	47.8 ± 12.6 a	0.5 ± 2.5 a	14.3 ± 4.8	12.2 ± 5.5	9.7 ± 15.9 c	0.2 ± 1.b
0.75× MAC (6)	69.0 ± 18.8 a	3.2 ± 12.9 a	15.4 ± 5.4	18.5 ± 7.3	33.2 ± 35.5 b	0.1 ± 0.2 b
Recovery (7)	54.1 ± 29.1 a	11.9 ± 13.0 a	13.8 ± 9.6	13.5 ± 8.6	45.6 ± 37.2 a	9.7 ± 13.7 a

**Table 2:** Cardiovascular parameters and maximum tolerance to the electrical stimulation (Hz) of six dogs subjected to isoflurane from awake (Phase 0) to induction (Phase 1), and then to profound anesthesia (Phase 2, 2.5× MAC), deep anesthesia (Phase 3, 2× MAC), surgical plane (Phase 4, 1.5× MAC), light anesthesia (Phase 5, 1× MAC), minimum anesthesia (Phase 6, 0.75× MAC), and recovery (Phase 7). Data are presented as mean ± SD. The * indicates that there is a significant difference (*p* < 0.01) between each treatment phase. Within the column, different letters (a, b, c, d, and e) indicate significant differences. The same letter indicates no significant difference.

Phase	End-Tidal * Isoflurane Concentration (%)	HR (bpm) *	SBP * (mmHg)	MBP * (mmHg)	DBP * (mmHg)	* Tolerance to Electrical Stimulation (Hz, Maxim 900 Hz)	Depth Score (1—Deep, 5—Light)
Awake baseline (0)	NA	97.1 ± 20.8 a	156 ± 23.4 a	108.5 ± 16.7 a	90.9 ± 18.4 a	NA	NA
Isoflurane induction (1)	2.8 ± 1.3 a	119.5 ± 26.2 b	91.7 ± 16.2 b	60.0 ± 4.4 b	48.3 ± 5.5 b	NA	3.0 ± 0.0
2.5× MAC (2)	3.3 ± 0.1 b	121.4 ± 13.6 b	67.7 ± 20.9 c	45.6 ± 16.4 c	41.3 ± 17.9 c	888.0 ± 33.2 a	2.1 ± 0.7
2× MAC (3)	2.6 ± 0.1 a	120.9 ± 12.4 b	76.9 ± 14.1 c	50.4 ± 7.4 c	40.6 ± 6.9 c	888.6 ± 32.3 a	2.3 ± 0.7
1.5× MAC (4)	1.9 ± 0.1 c	126.8 ± 15.2 b	116.1 ± 8.9 d	78.8 ± 8.9 d	60.0 ± 7.1 d	880.6 ± 62.4 a	2.8 ± 0.6
1× MAC (5)	1.3 ± 0.1 d	132.3 ± 19.3 c	139.2 ± 12.4 e	99.8 ± 13.2 e	82.1 ± 11.0 e	553.7 ± 234.1 b	3.7 ± 0.6
0.75× MAC (6)	0.9 ± 0.1 e	139.2 ± 19.2 c	156 ± 16.1 a	114.7 ± 13.6 a	98 ± 13.3 a	NA	4.0 ± 0.0
Recovery (7)	0.5 ± 0.2 e	153.6 ± 19.3 d	162.7 ± 21.2 a	122.9 ± 25.9 a	105 ± 26.4 a	NA	4.7 ± 0.5

## Data Availability

The data are contained within the article.

## Appendix A

**Table A1:** Subjective anesthetic depth score in dogs. The palpebral reflex was assessed with a cotton swab and the corneal reflex was assessed with sterile saline drops on the corneal surface via a syringe [15].

Anesthetic Plane	Depth Score	Pupil Size	Globe Position	Eye Reflex (P: Palpebral; C: Corneal)	Heart Rate, Blood Pressure, Breathing	Third Eyelid Position	Jaw Tone, Body/Limb Movement, or Swallowing
Very light	5	Large or small, moving	Central	P: active C: active	Increased, spontaneous breathing attempts	Retracted	Strong
Medium–light	4	Large or small	Central or ventromedial	P: mildly depressed C: mildly depressed	Increased, spontaneous breathing attempts	Retracted or partially prolapsed	Medium
Medium	3	Small or medium	Ventromedial	P: markedly depressed C: mildly depressed	Normal	Completely prolapsed	None or barely
Medium–deep	2	Medium	Central	P: absent C: markedly depressed	Normal or depressed	Completely or partially prolapsed	None
Very deep	1	Large	Central	P: absent C: absent	Depressed	Partially depressed	None
